# Carbon-Supported Pd and PdFe Alloy Catalysts for Direct Methanol Fuel Cell Cathodes

**DOI:** 10.3390/ma10060580

**Published:** 2017-05-25

**Authors:** Luis M. Rivera Gavidia, David Sebastián, Elena Pastor, Antonino S. Aricò, Vincenzo Baglio

**Affiliations:** 1 Departamento de Química, Instituto de Materiales y Nanotecnología, Universidad de La Laguna, Avda. Astrofísico Francisco Sánchez s/n, La Laguna, Santa Cruz de Tenerife 38200, Spain; lriverag@ull.edu.es; 2 Istituto di Tecnologie Avanzate per l’Energia “Nicola Giordano”, CNR. Via Salita S. Lucia sopra Contesse 5, Messina 98126, Italy; sebastian@itae.cnr.it (D.S.); arico@itae.cnr.it (A.S.A.)

**Keywords:** palladium, iron, direct methanol fuel cell, oxygen reduction reaction, methanol tolerance

## Abstract

Direct methanol fuel cells (DMFCs) are electrochemical devices that efficiently produce electricity and are characterized by a large flexibility for portable applications and high energy density. Methanol crossover is one of the main obstacles for DMFC commercialization, forcing the search for highly electro-active and methanol tolerant cathodes. In the present work, carbon-supported Pd and PdFe catalysts were synthesized using a sodium borohydride reduction method and physico-chemically characterized using transmission electron microscopy (TEM) and X-ray techniques such as photoelectron spectroscopy (XPS), diffraction (XRD) and energy dispersive spectroscopy (EDX). The catalysts were investigated as DMFC cathodes operating at different methanol concentrations (up to 10 M) and temperatures (60 °C and 90 °C). The cell based on PdFe/C cathode presented the best performance, achieving a maximum power density of 37.5 mW·cm^−2^ at 90 °C with 10 M methanol, higher than supported Pd and Pt commercial catalysts, demonstrating that Fe addition yields structural changes to Pd crystal lattice that reduce the crossover effects in DMFC operation.

## 1. Introduction

High energy conversion systems are required to satisfy global consumption demand. Fossil fuel usage is causing gradual environmental deterioration due to CO_2_ emission into the atmosphere [1], and consequently, the search for novel substitute sources is vital. Polymer electrolyte membrane fuel cell (PEMFC) technology is an innovative alternative to efficiently produce cleaner energy. Among these, direct methanol fuel cells (DMFCs) are supplied with methanol solutions as fuel at the anode. In contrast to other fuels derived from petroleum and organic sources, methanol has the largest oxidation electro-activity [1,2]. Commonly, DMFCs are used in portable systems due to their versatility and easy re-fueling and because they are very appealing from economic and environmental points of view [2,3,4]. However, a few technical barriers restrict DMFC commercialization; the main concerns are (i) the slow electro-kinetics of methanol oxidation and oxygen reduction at the anode and cathode, respectively, at low temperatures, forcing the use of platinum-based catalysts [3]; (ii) membrane degradation; (iii) performance loss due to methanol crossover caused by the low tolerance to permeated methanol of the cathodic catalysts commonly used (Pt) [4,5]. Nevertheless, all the other components, such as the cell housing, bipolar plates, gaskets and stack auxiliaries, also contribute to DMFC durability issues [3,5]. The methanol crossover above refers to the permeation of methanol through the electrolyte from the anode to the cathode, which causes a substantial performance decrease due to the formation of a mixed potential at the cathode [6,7,8,9]. Great efforts have been made to overcome these problems, particularly to replace the platinum based catalysts with non-noble metals [10] or other cheaper noble metals to be used as methanol tolerant cathodes, such as palladium [11]. It is well known that palladium-based catalysts present a good methanol tolerance as fuel cell cathodes [12] since the methanol oxidation process is negligible on Pd in acid media [13]. In this context, there are some recent investigations explaining the methanol tolerance of Pd-based cathodes; e.g., DFT studies of methanol adsorption on a Pt and Pd single layer composed of thirteen atoms showed how platinum distorts the molecular structure of the adsorbed methanol and favors its deprotonation in the first step of methanol oxidation process [14]. However, the deprotonation of methanol on palladium is kinetically and thermodynamically not favored, which is attributed to the Pd and Pt d-orbital extension difference. It is also known that Pd alloys with several 3d transition metals present higher oxygen reduction reaction (ORR) electro-activity than pure Pd [15]. Metals like Cu, Fe [13,15], Ni, Cr, Co [16,17], have been deeply studied for improving ORR activity and methanol tolerance and, therefore, minimizing the crossover effects [13,15,16,17]. On the other hand, core-shell palladium nanoparticles represent a good alternative as a cathodic material [18]. In this sense, Jia X. Wang et al. showed how a Pt monolayer growth (shell) on Pd with PdCo as the core exhibited prominent activity enhancement compared to Pt nanoparticles, due to strain and surface contraction effects. Likewise, Ru@Pd-Pt compared to Pd@Pt core shell structures, with low metal loading ~0.3 mg·cm^−2^ at the PEMFCs cathode side, exhibited a high performance attributed to Ru core electronic interaction with the Pd-Pt alloy shell [19]. It is often reported that Pd-d electron filling from another transition metal promotes a decrease of the density of states (DOS) due to the hybridization of the d-band of Pd by the incorporation of a second electropositive metal; thus, the adsorbed oxygen bonds are weakened and the dissociation mechanism is more feasible [15,16]. 

Previously, we proposed a trimetallic PdFeIr/C catalyst as a novel DMFC cathode due to its high tolerance toward methanol crossover effects, as demonstrated both in rotating disk electrode (RDE) experiments in half-cell configuration and at the cathode of a DMFC in single-cell tests. This was attributed to the surface composition rich in iron and iridium oxides. Nevertheless, a PdFe/C (without iridium) exhibited a similar behavior than PdFeIr/C in the kinetic region of ORR polarization curves in RDE experiments [13]. Despite PdFeIr/C’s low Pd content, the presence of iridium results in a cost increase compared to bimetallic PdFe catalyst for equivalent Pd loadings in the electrode. From that point of view, this modification represents inherent cost savings and, for this reason, we have decided to study the DMFC performance of the bimetallic catalyst in depth in this work.

## 2. Results

### 2.1. Physico-Chemical Characterization of the Synthesized Materials

PdFe/C and Pd/C were synthesized as described in the experimental section of this paper. The real atomic composition of the prepared catalysts, together with a commercial one (Pd/C ETEK), was determined by energy-dispersive X-ray (EDX) technique, and the data are depicted in Table 1. The results confirm that the metal loading of in-house prepared catalysts (Pd/C and PdFe/C) is close to 20 wt. % and the Pd:Fe atomic ratio is close to 3:1 for the PdFe/C catalyst. STEM images (Figure S1) show a homogeneous distribution of Pd and Fe for the PdFe/C catalyst confirming the atomic ratio from EDX (Figure S2). 

**Figure 1:**
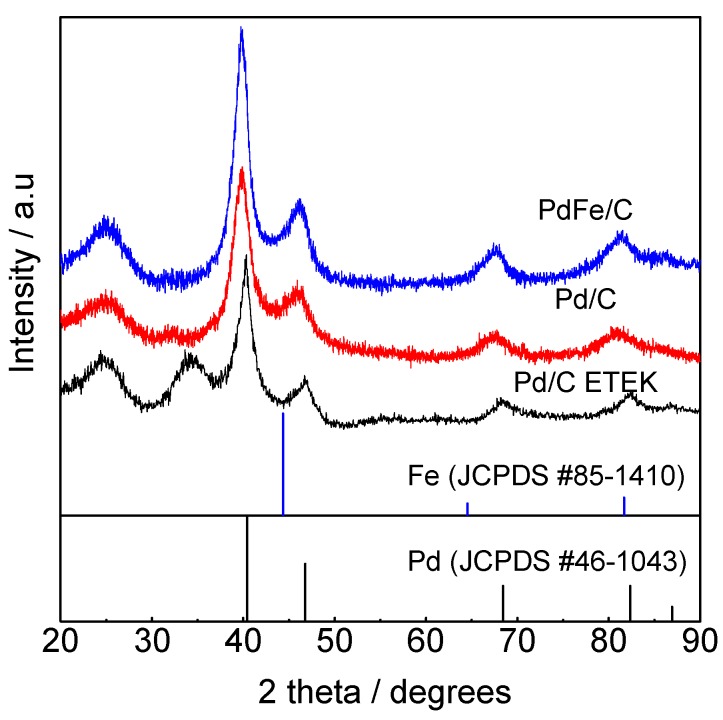
XRD patterns of Pd-based catalysts.

Figure 1 compares the X-ray diffractograms for Pd-based materials. The first peak at ca. 2θ = 25° for all diffractograms corresponds to (002) reflection of carbon support [19,20,21]. The other displayed five peaks are characteristic of the face-centered-cubic (fcc) crystalline structure of palladium (JCPDS #46-1043) [13,19], ascribed to the (111), (200), (220), (311) and (222) reflections. A pronounced diffraction peak close to 35° was observed in the Pd/C commercial catalyst, which can be attributed to the main reflection of Pd oxide-hydroxide species (PdO·xH_2_O, JCPDS #09-0254) [22]. A shift of Pd-related diffraction peaks of Pd/C and PdFe/C to lower 2𝛳 values than the benchmark Pd/C-ETEK was also observed, which is confirmed by the lattice parameter enlargement for Pd/C and PdFe/C catalysts (Table 1). The slight contraction in the latter (3.922 Å) compared to Pd/C (3.949 Å) can be ascribed to the alloy formation due to Fe insertion into the Pd crystalline structure [13,23,24]. Pd fcc structure has been reported to change when incorporating some transition elements with different atomic radius inside the Pd structure (e.g., Co [16], Ag [25], Ni [16,17], Fe [17,24]). It is known that the shift in XRD reflections can be attributed to alloy formation, increasing or decreasing according to the second metal atom size, producing a contraction or enlargement of the lattice parameter [17]. In our case, the reduction with sodium borohydride appears to promote a contraction of the Pd unit cell in the PdFe catalyst. The crystallite sizes, calculated by the Scherrer equation using Pd (220) peak, and the interplanar spacing, are reported in Table 1. The Pd crystallite size of Pd/C and PdFe/C catalysts are very similar [13], while the commercial Pd/C presents a slightly lower value. 

**Figure 2:**
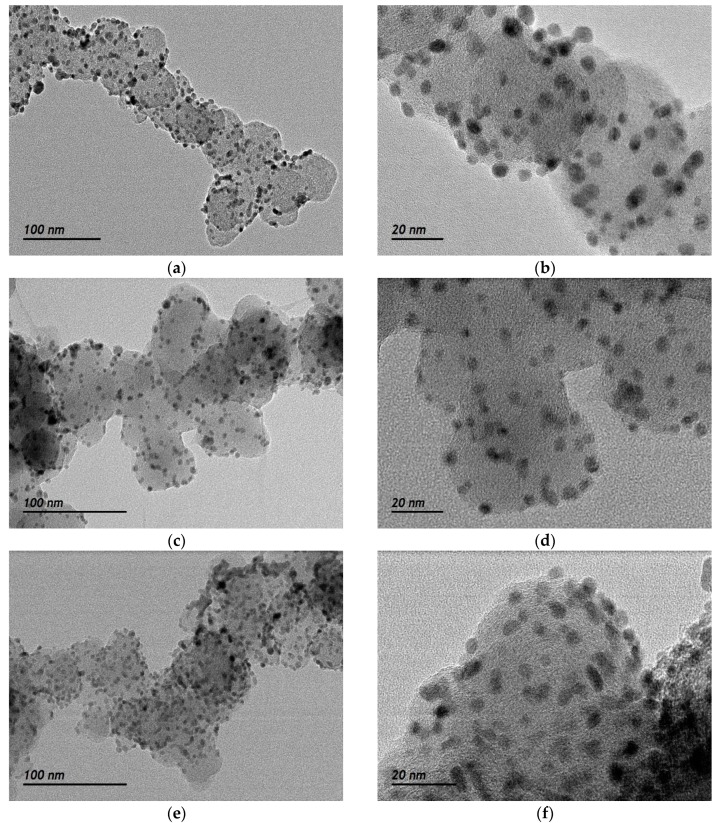
TEM images of (**a**) Pd/C at low magnification; (**b**) Pd/C at high magnification; (**c**) PdFe/C at low magnification; (**d**) PdFe/C at high magnification; (**e**) Pd/C-ETEK at low magnification; (**f**) Pd/C-ETEK at high magnification.

**Figure 3:**
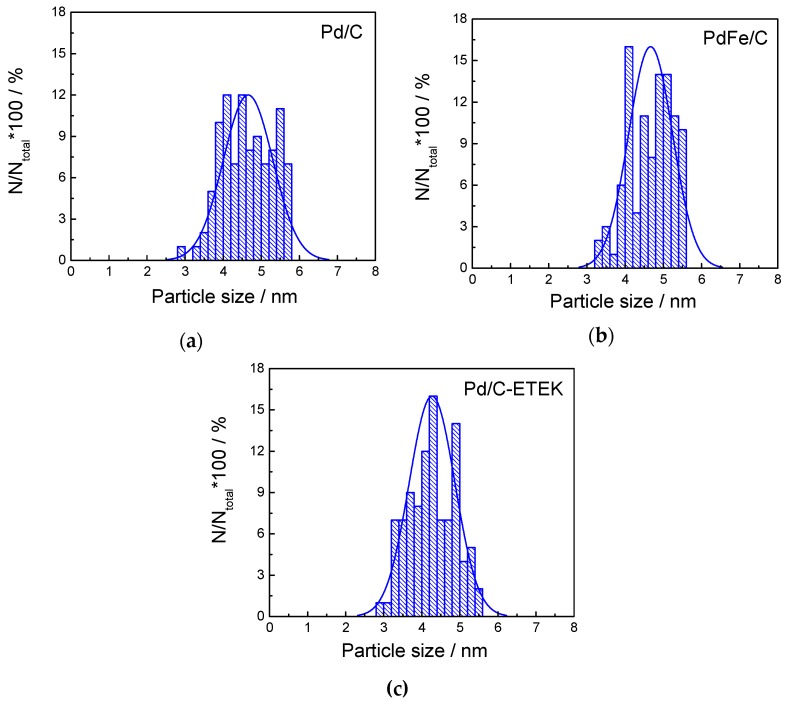
Particle size distribution histograms from TEM images of (**a**) Pd/C; (**b**) PdFe/C; (**c**) Pd/C-ETEK.

**Figure 4:**
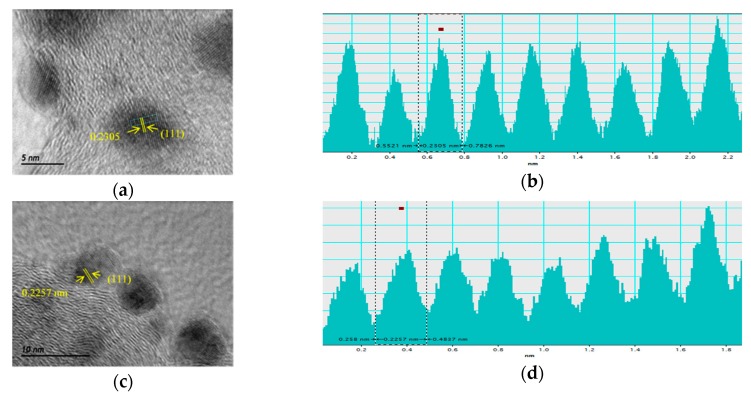

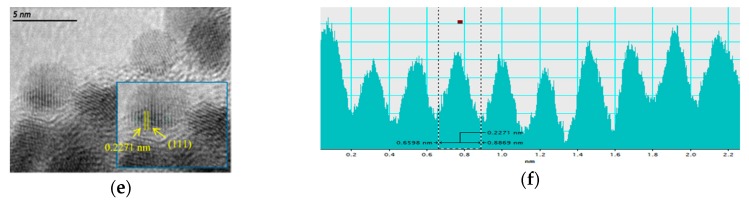
Analysis of interplanar spacing measurement from HRTEM images (**a**,**c**,**e**) for (**a**,**b**) Pd/C, (**c**,**d**) PdFe/C, (**e**,**f**) Pd/C-ETEK.

The Pd nanoparticles sizes and dispersion on the carbon support were evaluated by transmission electron microscopy (TEM). Figure 2 depicts the TEM micrographs at different magnifications for Pd/C, PdFe/C and Pd/C-ETEK. Spherical Pd-based nanoparticles with homogenous distribution on the carbon support were observed for all the catalysts. The particle size histograms (Figure 3) indicate slightly different distributions. The average particle sizes determined from TEM (Table 1) are higher than XRD crystallite sizes but with the same trend. The Gaussian fit of the particle size distributions (continuous line in Figure 3) reveals some agglomeration for both Pd/C and PdFe/C, since the distributions are asymmetrical with some more particles being larger than the average sizes. These results are in agreement with previous works where the borohydride method and other similar synthesis methods for carbon-supported nanoparticles have been used [26,27,28]. High resolution TEM micrographs (Figure 4) show ordered equidistant fringes for Pd with preferential orientation, as indicated in the images. The line profiles analysis of these images suggests the (111) planes are predominant [29,30]. Interestingly, the interplanar spacing decrease (Table 1) reflects the modification of the fcc Pd lattice by intercalation of Fe in the PdFe/C catalyst [24], also as confirmed by XRD analyses. Nevertheless, the interplanar spacing values determined by TEM analysis do not follow the same trend as those from XRD patterns since it is slightly lower for Pd/C-ETEK than for the PdFe/C catalyst.

**Figure 5:**
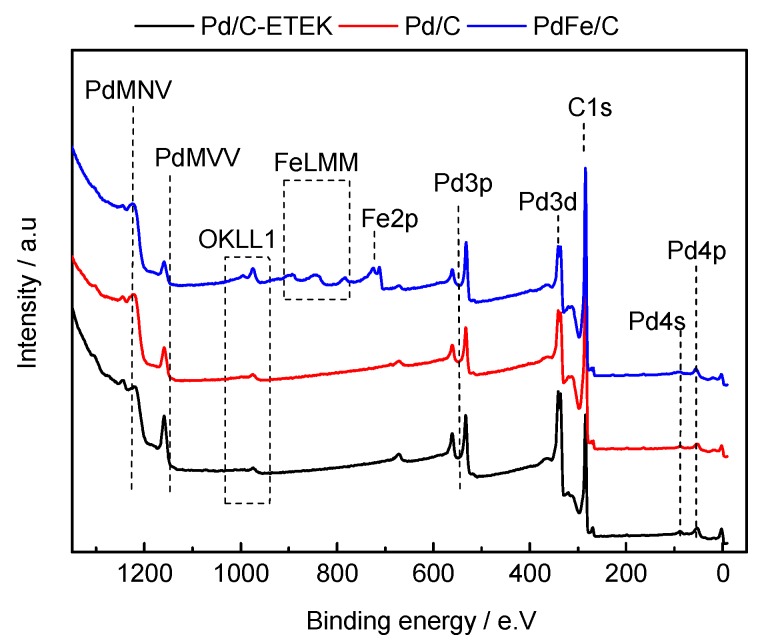
X-ray photoelectron survey spectra of Pd-based catalysts.

X-ray photoelectron spectra (XPS) of the Pd-based catalysts are shown in Figure 5. XPS analysis allowed determining the surface composition of each material. At a glance, it is possible to identify the photoelectron lines associated with metal composition in each catalyst: Pd 3d, Fe 2p, C 1s and O 1s core level were clearly identified [13,31]. As well, Auger lines localized at high binding energy values of several elements can be seen. In this sense, different Auger contributions can be observed, as indicated in the figure: Pd MNV, Pd MVV, Fe LMM and O KLL. As can be seen, the O KLL Auger lines in the PdFe/C have a significant intensity compared with the other two catalysts, which is attributed to iron oxides. The oxide amount present on the catalysts surface and the binary alloy produces a superficial metallic Pd rise. On the other hand, Pd/C-ETEK surface composition is almost the same as Pd/C (Figure S3 and Table S1). Atomic compositions estimated by XPS have already been reported [13]. 

### 2.2. Electrochemical Performances of Pd-Based Catalysts in DMFC

**Figure 6:**
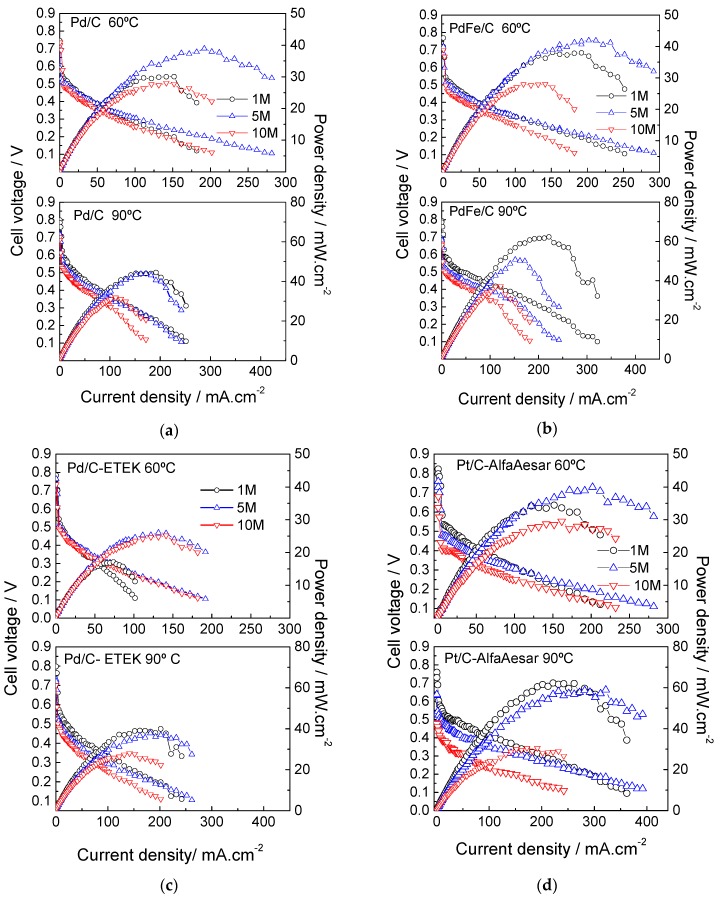
DMFC performances (polarization and power density curves) at 60 °C (top panel) and 90 °C (bottom panel) of (**a**) Pd/C; (**b**) PdFe/C and (**c**) Pd/C-ETEK; (**d**) Pt/C-AlfaAesar.

The electrochemical behavior of all homemade materials in this work was studied at the cathode side of a DMFC and compared with commercial catalysts: the Pd/C-ETEK and a Pt/C (Alfa Aesar, 2.4 nm crystallite size). Methanol concentration and temperature effects have been studied. Figure 6a–d shows the polarization and power density curves with 1 M, 5 M and 10 M methanol concentration at two different temperatures (60 and 90 °C) for the MEAs based on Pd/C, PdFe/C, Pd/C-ETEK catalysts and Pt/C-AlfaAesar, respectively. The anode (PtRu black) and membrane (Nafion^®^ 115) were kept constant in all MEAs. 

In general, a substantial performance improvement was observed with increasing temperature for all MEAs, mainly due to methanol oxidation and oxygen reduction kinetics enhancement [32,33]. All polarization curves exhibit the typical sharp potential decay at low current density associated with the activation process of both MOR and ORR, followed by a linear decay associated with ohmic losses. Some cases present an additional source of voltage decay at high current density, attributed to mass transport constraints at the electrodes. Regardless of the operating temperature, the cell voltage in the activation zone generally decreases with the increase of methanol concentration, due to the detrimental effect of methanol crossover. This is more exacerbated in the Pt/C cathode-based MEA (Figure 6d). Whereas, the voltage decrease with current associated to methanol crossover is much lower with the Pd-based cathodes, being almost negligible (few millivolts) at 60 °C. The methanol permeation rate, at a determined set of operating conditions, such as temperature and anode fuel concentration, relies mostly on membrane characteristics (composition, thickness, etc.). Considering that the tested MEAs differ only in the cathode catalyst layer, variations of potential in the low current region with methanol concentration may be attributed to methanol tolerance characteristics of the cathode. As a consequence, the few millivolts voltage decay with Pd cathodes is a clear indication of the higher tolerance to the presence of methanol of the Pd catalysts compared to Pt. At a low methanol concentration (1 M) and 60 °C, the open circuit potential (OCP) values follow this order: PtC/-AlfaAesar > Pd/C-ETEK > PdFe/C > Pd/C. Under these working conditions, the crossover effects are not so detrimental [7,9]; thus, OCP usually increases when the temperature rises for low methanol concentrations (i.e., 1 M) [9,33,34]. However, the methanol permeation rate increases with temperature [35]. Concerning the maximum current density, Pd-based cathodes exhibit similar (PdFe/C) or lower values than the MEA equipped with a Pt catalyst. As well-known, the methanol crossover decreases with the increase of current density. Pt cathodes are more active towards the ORR than Pd in the absence of permeated methanol, and, as a consequence, moving to the low voltage region (low efficiency) leads to lesser extent of methanol adsorption on Pt. 

Feeding an intermediate methanol concentration (5 M) at the same temperature (60 °C) results in a lower cell voltage at low current density (including OCP) compared to the performances with 1 M methanol. The effect of methanol crossover is further accentuated when the single cell is fed with 10 M methanol. The in-house prepared Pd/C catalyst showed the highest tolerance to crossover effects in terms of voltage, followed by Pd/C-ETEK and PdFe/C, whereas Pt/C-AlfaAesar presented the highest OCP decay of 140 mV associated with the methanol concentration increase from 1 to 10 M [9,32,33]. This corroborates the low methanol tolerance for Pt-based cathodes and the great advantage of using Pd-based materials [13,23,24,25]. It is of great interest to reduce or, better, suppress the use of Pt for this application, favoring market access to DMFCs. In this sense, palladium-enriched catalysts for DMFC cathodes have been designed and studied by several authors [11,12,13,14,15,16,17,18,23,24,25]. Our current results are comparable to what has been reported by Choi et al., where a palladium-rich catalyst (Pd_19_Pt_1_/C, 2.8 nm from XRD) shows relatively larger methanol tolerance compared to Pt/C at 70 °C and 6 M methanol concentration. However, the OCP values reported are lower than those achieved in this study [34]. The same applies to Pd_3_Pt/C (5 nm) catalysts described by Li et al. [36], which were synthesized through a modified polyol method, where OCP values at 1 M methanol concentration and 75 °C are lower compared to those presented here for Pd-based catalysts at the same methanol concentration and 90 °C. 

**Figure 7:**
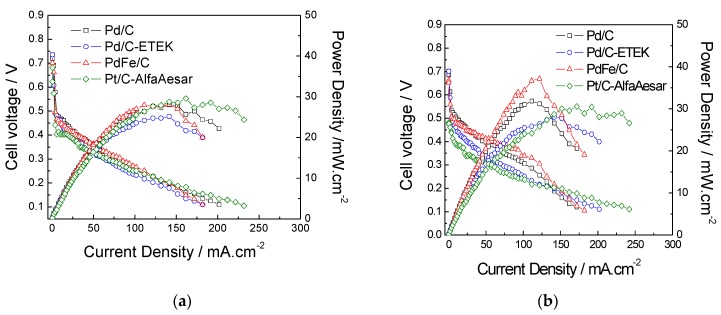
DMFC performance compared at 10 M of methanol concentration and at (**a**) 60 °C and (**b**) 90 °C.

A comparison of the polarization and power density curves obtained with 10 M methanol at 60 °C (Figure 7a) shows the influence of high methanol concentration on the performance of different Pd-based cathodes. At low current density (<50 mA·cm^−2^), the best performances were attained with Pd/C and PdFe/C cathode-based MEAs, slightly better than the commercial Pd/C and all of them exceeding the performance of the commercial Pt/C. Conversely, at high current density, the polarization curve of Pt/C behaves slightly better than those of Pd-based catalysts, achieving similar values of maximum power density to the PdFe/C-related MEA. At a higher operating temperature (90 °C), the differences among different cathodes are exacerbated (Figure 7b). At low current density, in the activation region of the polarization curves, the detrimental effect of methanol crossover leads to a larger voltage decay in the commercial-based cathodes, being more dramatic in the Pt/C catalyst due to its worse methanol tolerance properties. The in-house prepared Pd/C and PdFe/C catalysts exhibit a much lower voltage drop in the low current density region. The maximum power density at 90 °C reached 37 mW·cm^−2^ at 0.35 V for the MEA based on PdFe/C at the cathode. The introduction of Fe seems to be beneficial to operate at high temperature and high methanol concentration (high energy density DMFCs).

**Figure 8:**
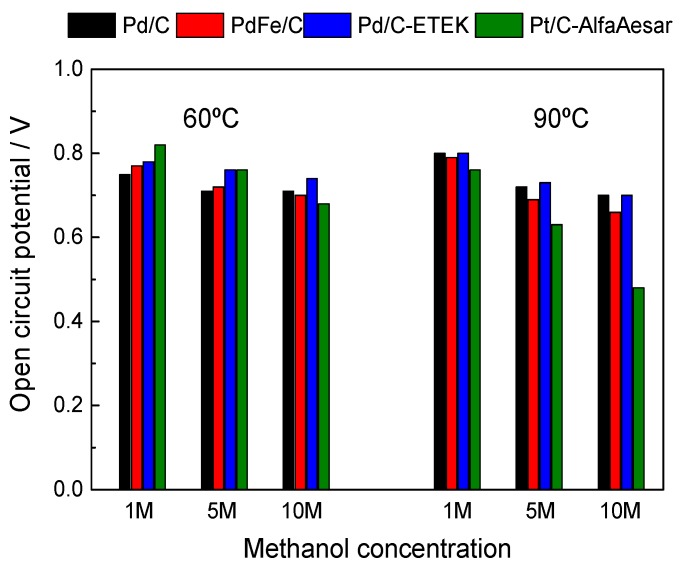
Dependence of open circuit potential of the MEAs studied with the working temperature and anode methanol concentration.

The OCP dependence with temperature and methanol concentration for the whole set of tested MEAs is displayed in Figure 8. The increase of methanol concentration produces, in general, a decrease of OCP for all the MEAs both at 60 °C and 90 °C, related to the increase of methanol permeation rate through the polymer membrane [35]. Pd-based cathodes exhibit the lowest decay in OCP, with 40 mV at 60 °C and 100 mV at 90 °C from 1 M to 10 M methanol. The PdFe/C catalyst is characterized by slightly higher OCP decay (70 mV and 130 mV at 60 °C and 90 °C, respectively) but much lower if compared to the Pt cathode MEA, which exhibited a loss of 140 mV at 60 °C and of 280 mV at 90 °C. The better OCP values when operating at high methanol concentration turn into an enhanced polarization behavior in the activation controlled zone of the DMFC, i.e., the high energy efficiency operating mode with a high energy density coming from the concentrated fuel.

**Figure 9:**
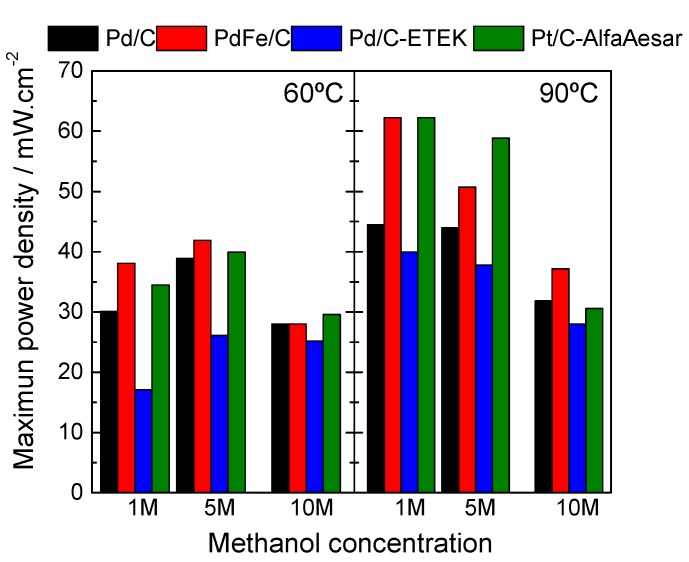
Maximum DMFC power densities as a function of cathode catalyst, working temperature (60 °C and 90 °C) and methanol concentration at the anode (1–10 M).

Figure 9 shows the maximum power density values as a function of methanol concentration for the investigated catalysts at 60 °C and 90 °C. The maximum power density occurs at relatively high current densities where the methanol crossover effects are less dramatic than at low current densities. The MEA equipped with the in-house prepared PdFe/C catalyst exhibited a similar or even better maximum power density than that equipped with the Pt/C catalyst, in particular at high methanol concentration and temperature (10 M and 90 °C), where methanol crossover constraints are more pronounced, while the commercial Pd/C catalyst presented the lowest power densities. In particular, the PdFe/C catalyst presented the highest performance and at 90 °C. Taking into account that palladium is cheaper than platinum, these results highlight palladium-based formulations as a reliable alternative to Pt for cost-effective and performing high-energy-density DMFC devices.

**Figure 10:**
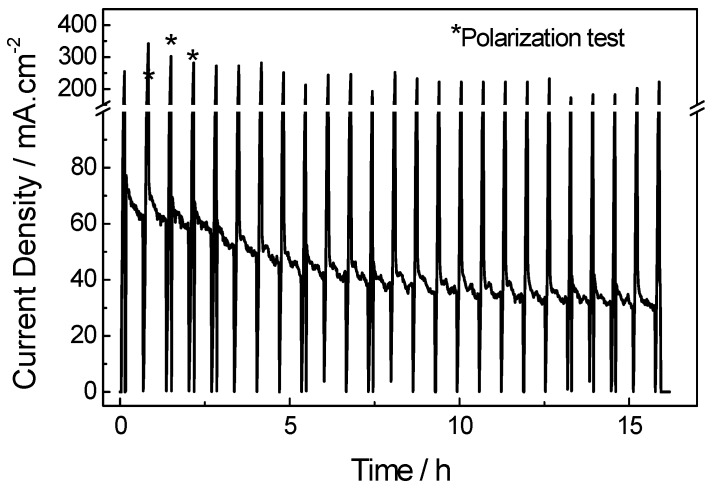
PdFe/C DMFC stability test at 0.4 V, 90 °C, 1 M methanol and fully humidified oxygen at flow rates of 2 and 100 mL·min^−1^, fed at the anode and cathode, respectively.

A DMFC stability test was carried out for the best performing cathode, PdFe/C, over 16 h at 0.4 V, with polarization curves every 20 min to evaluate cell performance, as shown in Figure 10. The current density peaks correspond to maximum current densities recorded during each polarization test at low voltage (close to 0.1 V). Initially, the steady-state current density tends to decrease with time during the first 8 h, with a decay rate of 4 mA·cm^−2^·h^−1^. The performance is partially recovered after every polarization curve, accounting for the reversible part of the total loss. Afterwards, the current decay is much slower (about 1 mA·cm^−2^·h^−1^). The maximum power density decreases from about 57 mW·cm^−2^ to 32 mW·cm^−2^, which represents 44% loss after 16 h of operation. In a previous work, a bimetallic catalyst based on PtFe was subjected to accelerated stress tests, and a partial loss of iron accounting for about 36% was observed [37], which may explain the irreversible part of the performance losses in our stability experiment.

## 3. Conclusions

Methanol-tolerant carbon-supported Pd-based catalysts (Pd/C and PdFe/C) were synthesized and investigated for DMFC cathode applications. The physico-chemical characterization including XRD, EDX, XPS and TEM confirmed the alloy formation for PdFe/C catalyst, surface composition and a good distribution of nanoparticles on carbon support. The DMFC studies in single cell configuration, using the Pd-based catalysts at the cathode side, revealed enhanced performance from PdFe/C compared with Pd and Pt commercial catalysts, especially when operating with high methanol concentration (10 M), where the methanol crossover effects are more significant. The use of palladium formulations at the cathode side appears very promising for DMFCs in applications where high energy capacity and prolonged duration are required. The better Pd tolerance of the presence of permeated methanol at the cathode results in improved oxygen reduction activity. More specifically, the MEA based on PdFe/C achieved 25% and 18% higher power density than those based on commercial Pd/C and Pt/C catalysts, respectively. The insertion of Fe into Pd nanoparticles aids the electrocatalytic activity for the oxygen reduction, which is ascribed to the electronic conjugation by superficial iron oxide and metallic Pd, accompanied by the interplanar spacing decrease detected by XRD and TEM analysis. The utilization of partially oxidized surface species based on abundant transition metal alloys with cheaper Pt-free metals like palladium offers a new path in the search for novel catalysts with high oxygen electro-reduction selectivity and low cost.

## 4. Materials and Methods

### 4.1. Catalyst Preparation

The in-house made 20 wt. % Pd/C and PdFe/C carbon-supported catalysts were prepared using the borohydride reduction method following the same procedure described in our previous paper [13]. Carbon supported commercial catalysts of 20% Pt/C and 30% Pd/C were purchased from Alfa Aesar and ETEK companies, respectively.

### 4.2. Physicochemical Characterization

Atomic composition and the metal loading of the catalysts were determined by X-ray energy dispersive (EDX) spectroscopy coupled to a scanning electronic microscope Jeol JSM 6300 with a silicon doped with lithium 6699 ATW detector applying 20 keV. X-ray diffractograms (XRD) were obtained using a Philips X-pert 3710 X-ray diffractometer with Cu Kα anodic radiation source. The Marquardt algorithm was used to fit the peak profile of the (220) reflection of the face centered cubic structure of Pd-based catalysts. The crystallite size was then calculated by applying the Debye-Scherrer equation. Transmission electron microscopy (TEM) images and STEM microanalysis were obtained with a Jeol 2100 (200 kV) microscope. Catalyst powders were dispersed in isopropyl alcohol using an ultrasonic bath. Then, a few drops of the dispersion were deposited on carbon film-coated Cu grids and dried with argon atmosphere. All images were studied with Gatan Microscopy Suite Software 2.0. The high-resolution X-ray photoelectron spectra (XPS) were collected on a ESCALAB 250 spectrometer equipped with dual aluminum-magnesium anodes, using a monochromatized Al Kα X-ray radiation (hν = 1486.6 eV) with a spot size of 650 µm. The spectrometer energy calibration was performed using the Au 4f_7/2_ and Cu 2p_3/2_ photoelectron lines. The spectra were collected in constant analyzer energy (CAE) mode, with pass energy of 20 eV and with an energy resolution of about 0.1 eV. For all the measurements, the pressure in the ultra-high vacuum analysis chamber was less than 9 × 10^−9^ mbar, avoiding the ejected photoelectron interact with gas molecules. In XPS data analysis all binding energies (BE) values were calibrated using C 1s peak at 284.6 V to take into account the chamber environment and the charge effect during the measurement. The XPS data interpretation was performed using standard data from Perkin-Elmer Corporation X-ray photoelectron spectroscopy handbook [31].

### 4.3. Electrochemical Studies

Commercial gas diffusion layers (GDLs, E-TEK) were used at both electrodes to prepare the membrane-electrode assemblies (MEAs). Concerning the cathode electrodes, homemade Pd/C and PdFe/C catalysts as well as commercial Pd/C (ETEK) and Pt/C (Johnson Matthey) were sprayed onto a GDL (GDL-LT, E-TEK). An adequate amount of catalysts powder was ultrasonically dispersed in a solution consisting of water and isopropyl alcohol (1:3) and Nafion^®^ ionomer (33 wt % in the catalytic layer). Subsequently, the resulting ink was sprayed onto the GDL with an atomizing gun connected to a compressed air supply. The spray-painting process is completed when achieving a Pd or Pt loading of 1 mg cm^−2^. On the other hand, the anodic electrodes consisted of PtRu (1:1) unsupported nanoparticles (Johnson Matthey) mixed with 15 wt % Nafion and deposited on the GDL (GDL-HT, E-TEK) using the doctor blade technique [20]. The Pt loading at the anode was 1 mg cm^−2^ in all MEAs. A Nafion^®^ 115 membrane was employed as the polymer electrolyte. MEAs were assembled using a hot-pressing procedure at 130 °C for 90 s, 30 kg-f·cm^−2^ using a hydraulic press equipped with heating plates. Afterwards, the MEAs were placed in a fuel cell test fixture of 5 cm^2^ of area. The single cell was then connected to a DMFC test station by Greenlight (Hydrogenics). The experiments were carried out by feeding aqueous methanol solutions with concentrations of 1 M, 5 M and 10 M to the anode compartment (2 mL·min^−1^), and fully humidified oxygen was fed to the cathode side (100 mL·min^−1^). The electrochemical response of the MEAs was studied at 60 °C and 90 °C, monitoring the temperature with a thermocouple embedded into the cathode endplate close to the MEA. The single cell performances were investigated under steady-state conditions. A 16 h DMFC short stability test was carried out for PdFe/C cathode catalyst. Cell operating conditions were 90 °C, 1 M methanol fed to the anode at 2 mL·min^−1^ and fully humidified O_2_ fed to the cathode at 100 mL·min^−1^. The cell potential was kept 0.4 V in intervals of 20 min with pauses to perform polarization curves. The open circuit potential (OCP) values were measured in steady state mode, about 15–20 min after starting to feed the cell with methanol solution and humidified oxygen. In the first few minutes after injection of fuel and oxidant, OCP increases quickly due to a dynamic overshoot and then slightly decreases to a constant potential as reported in other works [38,39]. Thus, OCP values measured in the steady-state condition are not influenced by transient phenomena related to hydrogen evolution/oxidation reactions. 

## Figures and Tables

**Table 1:** Physico-chemical properties of the Pd-based catalysts.

Catalysts	Crystallite Size ^1^ (nm)	Interplanar Spacing ^1^ (Å)	Lattice Parameter ^1^ (Å)	Particle Size ^2^ (nm)	Interplanar Spacing ^2^ (Å)	Pd:Fe Atomic Ratio ^3^ (at %)	Metal Loading ^3^ (wt %)
Pd/C	3.9	2.273	3.949	4.7 ± 0.7	2.305	-	20
PdFe/C	3.8	2.261	3.922	4.6 ± 0.5	2.257	76:24	19
Pd/C ETEK	3.4	2.241	3.883	4.3 ± 0.6	2.271	-	30

^1^ XRD, ^2^ TEM, ^3^ EDX.

## Supplementary Materials

The following are available online at http://www.mdpi.com/1996-1944/10/6/580/s1, Figure S1: STEM images at high magnification (200,000×) of PdFe/C catalyst. From left to right and from top to down, catalyst image, Pd mapping, Fe mapping, C mapping and O mapping, Figure S2: X-ray energy dispersive spectrum of PdFe/C catalyst, Figure S3: Pd 2d core level XPS spectrum of Pd/C commercial catalyst, Table S1: Relative areas (%) and binding energies (eV) from the deconvolution of Pd 2d XPS spectrum of Pd/C commercial catalyst.
