# Supplementary material for: Carbon-Supported Pd and PdFe Alloy Catalysts for Direct Methanol Fuel Cell Cathodes
Source: Materials (Basel). 2017 May 25;10(6):580. doi: 10.3390/ma10060580 (PMC5552173; doi:10.3390/ma10060580)
Supplement: Supplementary file 1 [file materials-10-00580-s001.pdf]

Supplementary Data

Carbon-Supported Pd and PdFe Alloy Catalysts for Direct Methanol Fuel Cell Cathodes

Luis M. Rivera Gavidia, David Sebastián, Elena Pastor, Antonino S. Aricò and Vincenzo Baglio

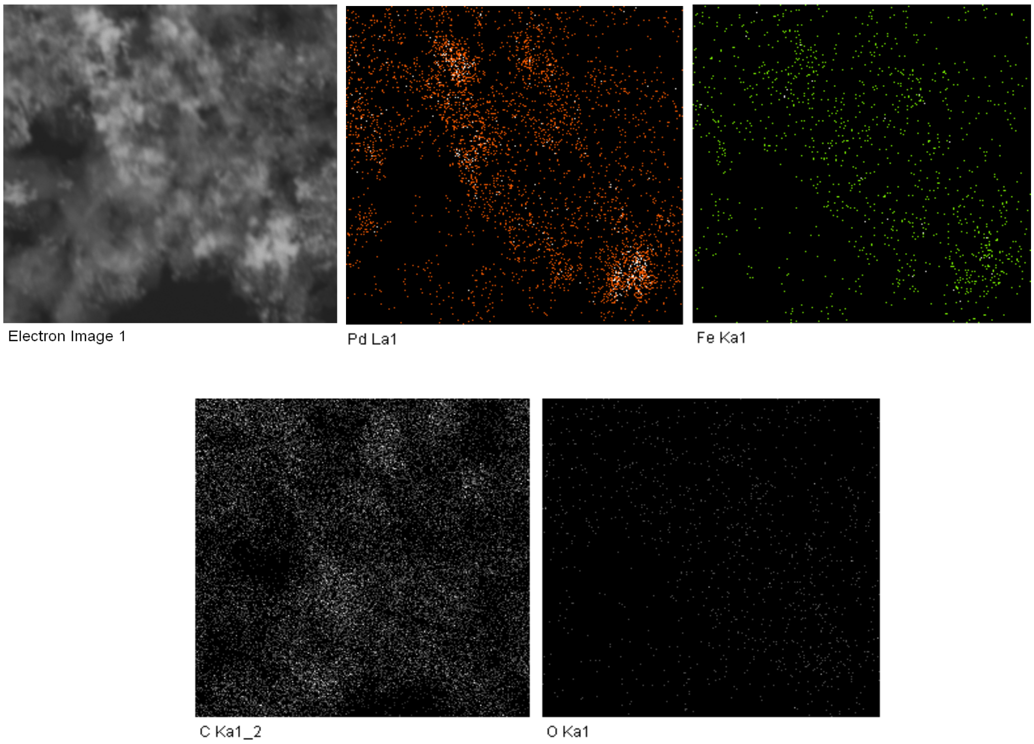

Figure S1. STEM images at high magnification (200000×) of PdFe/C catalyst. From left to right and from top to down, catalyst image, Pd mapping, Fe mapping, C mapping and O mapping.

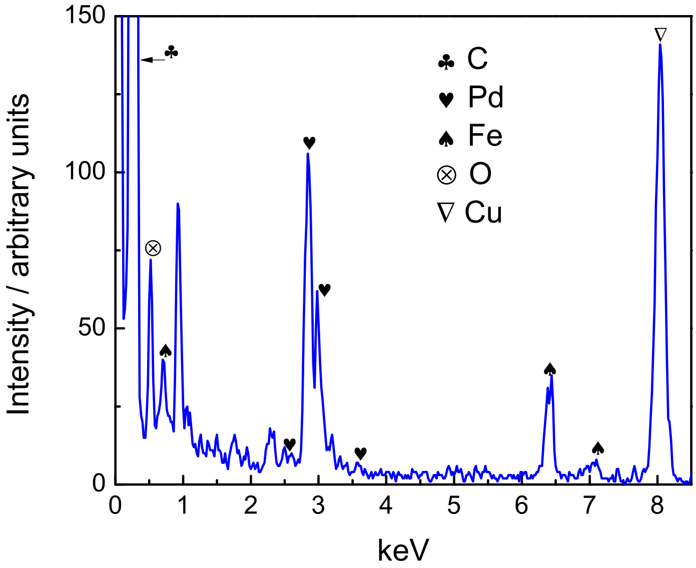

Figure S2. X-ray energy dispersive spectrum of PdFe/C catalyst.

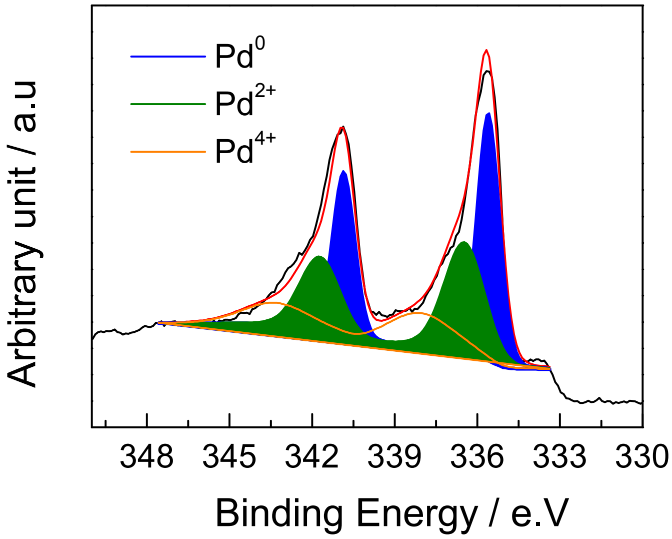

Figure S3. Pd 2d core level XPS spectrum of Pd/C commercial catalyst.

Table S1. Relative areas (%) and binding energies (eV) from the deconvolution of Pd 2d XPS spectrum of Pd/C commercial catalyst.

Pd^0	PdO/Pd^{2+}	PdO_2/Pd^{4+}
47.2 (335.6 eV)	35.8 (336.5 eV)	17 (338.1 eV)
